# Cycling Waveform Dependent Wake-Up and ON/OFF Ratio in Al_2_O_3_/Hf_0.5_Zr_0.5_O_2_ Ferroelectric Tunnel Junction Devices

**DOI:** 10.1021/acsaelm.2c01492

**Published:** 2023-03-10

**Authors:** Keerthana Shajil Nair, Marco Holzer, Catherine Dubourdieu, Veeresh Deshpande

**Affiliations:** † Helmholtz-Zentrum-Berlin für Materialien und Energie, Institute “Functional Oxides for Energy Efficient Information Technology”, Hahn-Meitner Platz 1, 14109 Berlin, Germany; ‡ Physical Chemistry, Freie Universität Berlin, Arnimallee 22, 14195 Berlin, Germany

**Keywords:** hafnium zirconium oxide, FTJ, wake-up, MFDM stack, neuromorphic

## Abstract

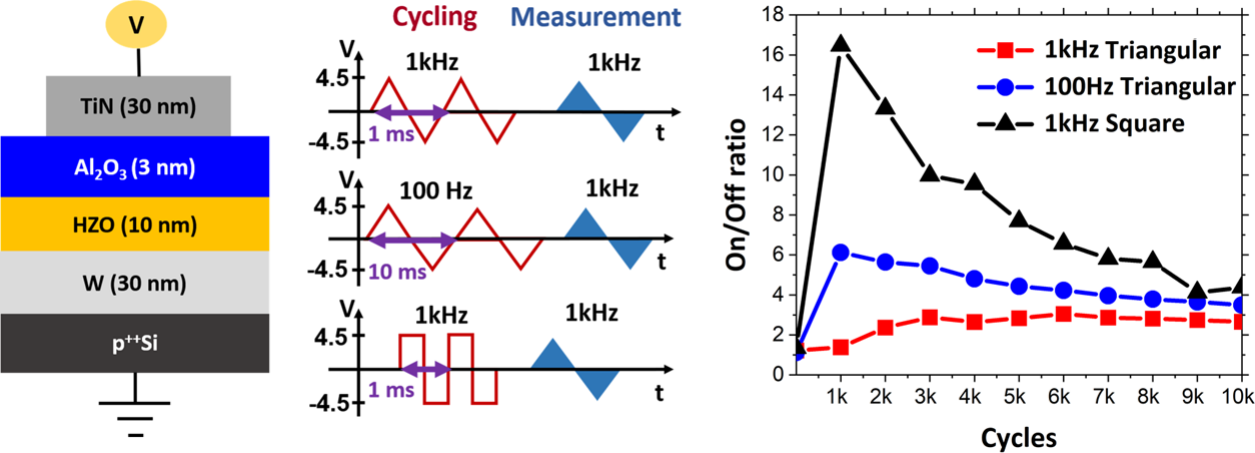

The wake-up behavior and ON/OFF current ratio of TiN–Al_2_O_3_–Hf_0.5_Zr_0.5_O_2_–W ferroelectric tunnel junction (FTJ) devices were investigated for different wake-up voltage waveforms. We studied triangular and square waves, as well as square pulse trains of equal or unequal voltage amplitudes for positive and negative polarities. We find that the wake-up behavior in these FTJ stacks is highly influenced by the field cycling waveform. A square waveform is observed to provide wake-up with the lowest number of cycles, concomitantly resulting in higher remnant polarization and a higher ON/OFF ratio in the devices, compared to a triangular waveform. We further show that wake-up is dependent on the number of cycles rather than the total time of the applied electric field during cycling. We also demonstrate that different voltage magnitudes are necessary for positive and negative polarities during field cycling for efficient wake-up. Utilizing an optimized waveform with unequal magnitudes for the two polarities during field cycling, we achieve a reduction in wake-up cycles and a large enhancement of the ON/OFF ratio from ∼5 to ∼35 in our ferroelectric tunnel junctions.

## Introduction

The discovery of ferroelectricity in hafnium oxide^1,2^ and its advancements over the past decade has fueled the development of complementary metal–oxide–semiconductor (CMOS)-compatible ferroelectric (FE) nonvolatile memory devices.^3^ The two-terminal simple architecture along with low power consumption, scalability, and high switching speed has made ferroelectric tunnel junctions (FTJs) a promising candidate for data storage and synaptic applications in neuromorphic computing.^4−6^ A conventional FTJ architecture consists of an ultra-thin ferroelectric (FE) layer sandwiched between two metal electrodes. To obtain a high tunneling current in such a device, the thickness of the ferroelectric has to be typically below 5 nm.^7^ Even though recent studies have shown stable ferroelectric polarization in the HfO_2_–ZrO_2_ solid solution system down to ∼1 nm,^8,9^ it is still quite challenging to stabilize high remnant polarization in such ultra-thin layers. As an alternative, it is possible to use metal–ferroelectric (FE)–dielectric (DE)–metal stack for FTJ devices.^10,11^ This architecture can provide a high ON/OFF ratio with a thick FE layer (typically around 10 nm). In the M–FE–DE–M stack, the tunneling of charge carriers occurs across the DE layer, whose thickness is typically on the order of 1–4 nm.^4,11,12^ The tunneling current across the dielectric is directly correlated to the polarization in the FE layer.^13,14^ It is well known that in Hf_0.5_Zr_0.5_O_2_ (HZO) ferroelectric layers, high remnant polarization develops after electric field cycling, known as the “wake-up” behavior. It is considered to originate either from retribution of oxygen vacancies,^15,16^ phase transformation during field cycling,^17,18^ or electron trapping.^19^ Although the wake-up effect in metal–HZO–metal capacitor structures has been extensively investigated,^19−23^ the effect of electric field cycling parameters has not been considered thoroughly. In particular, the understanding of the impact of electric field cycling parameters on device characteristics is crucial in FTJ memory and synaptic devices, as it has significant implications in circuit applications. A study has only recently addressed the cycling scheme in TiN/Al_2_O_3_/HZO/TiN bilayer FTJ devices.^24^ The complexity of the circuits necessary to wake up the FTJ stack depends on the waveform required. Additionally, for neuromorphic applications, the number of resistance states achievable depends on the ON/OFF ratio of the FTJ, which correlates strongly with the polarization wake-up resulting from the electrical cycling. Furthermore, the polarization state stability in bilayer devices is impacted by charge traps near the FE–DE interface.^25^ The role of the charge traps during wake-up has not been considered in bilayer FTJ stacks so far. Therefore, it is essential to investigate the impact of the voltage waveform on the wake-up behavior as well as the resultant electrical characteristics of the FTJ devices. Such a study will allow providing guidelines for the efficient electrical programming of the devices for neuromorphic applications and will also shed light on the intrinsic mechanisms in the FTJ stacks.

In this work, we investigate the impact of the voltage waveform on the wake-up behavior of TiN–Al_2_O_3_–Hf_0.5_Zr_0.5_O_2_–W devices. The effect of various cycling waveforms on the polarization of the HZO layer in the stack and on the ON/OFF ratio is first studied. We show that the wake-up behavior and memory characteristics are largely impacted by the type of waveform applied during the field cycling operation. We then optimize the wake-up cycling waveform to reduce the number of wake-up cycles needed and show an enhancement of the ON/OFF ratio up to ∼35.

## Experimental Details

**Figure 1:**
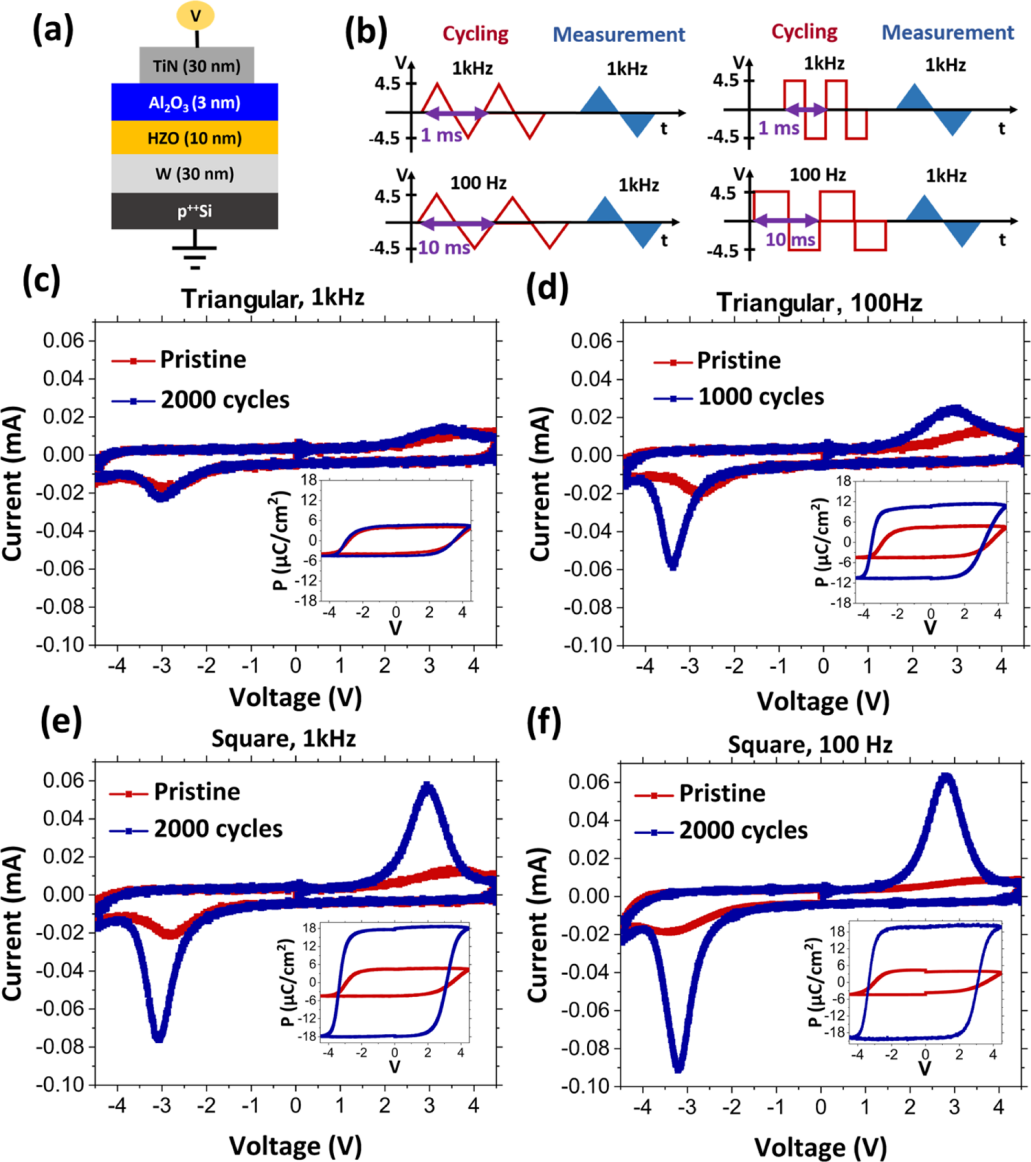
(a) Schematic of the TiN–Al_2_O_3_–Hf_0.5_Zr_0.5_O_2_–W FTJ devices under study. (b) Wake-up field cycling and measurement sequence corresponding to four different cycling waveforms: 1 kHz, 100 Hz triangular and 1 kHz, 100 Hz square. *I*–*V*, and *P*–*V* measurements are performed with a triangular waveform with a frequency of 1 kHz and a voltage of ±4.5 V after field cycling. *I*–*V* measurements for 1 kHz, 100 Hz triangular are shown in (c) and (d) and for 1 kHz, 100 Hz square in (e) and (f). All curves show a pristine state and after wake-up cycles. The insets in (c)–(f) show the PUND sequence *P*–*V* measurements.

The TiN–Al_2_O_3_–Hf_0.5_Zr_0.5_O_2_–W FTJ stack investigated in this study is fabricated with W as the bottom electrode and TiN as the top electrode, as shown in Figure 1a. First, the W bottom electrode with a thickness of 30 nm was sputter-deposited on a p++-doped Si wafer (resistivity ∼0.005 Ω cm) at room temperature. Native SiO_2_ was removed from the Si wafer surface by etching it in diluted HF prior to W deposition. After the deposition of the bottom electrode, 10 nm of HZO and 3 nm of Al_2_O_3_ were deposited by atomic layer deposition in an OXFORD FlexAl chamber at 250 °C using TEMA-Hf, TEMA-Zr, and TMA precursors separately. Water was used as a coreactant for oxidation. The TiN top electrode with a thickness of 30 nm was then deposited by sputtering at room temperature. The crystallization of the HZO layer to obtain the ferroelectric phase was carried out by rapid thermal annealing (RTA) of the whole stack at 400 °C for 120 s in N_2_ ambient. After the RTA, square pads of 95 μm × 95 μm areas were patterned in the top TiN electrode layer by first performing photolithography and liftoff of the Ti (30 nm)/Au (100 nm) bilayer followed by TiN etching using SC1 solution at 50 °C. The low-temperature crystallization of HZO films makes the FTJ devices CMOS back-end-of-line compatible and enables three-dimensional (3D) integration with CMOS circuits.^12^

The electrical characterization of the devices consisted of (a) ferroelectric polarization switching characterization through current–voltage (*I*–*V*) and polarization–voltage (*P*–*V*) measurements with the PUND sequence and (b) tunneling electroresistance characterization by first switching polarization in either up (polarization pointing toward Al_2_O_3_; “set” operation) or down (polarization pointing away from Al_2_O_3_; “reset” operation) direction with a voltage pulse followed by DC current measurement (“read” operation) with a constant voltage of −1.6 V (lower than the coercive voltage). The DC read operation was performed after each switching pulse to measure the ON and OFF currents. The *I*–*V* and *P*–*V* measurements were carried out with a commercial ferroelectric test system (Radiant Precision Multiferroic II). The same system was used for reset, set, and constant voltage read operations.

## Results and Discussion

The wake-up behavior of TiN–Al_2_O_3_–Hf_0.5_Zr_0.5_O_2_–W FTJ devices was first investigated with four different field cycling waveforms: 1 kHz, 100 Hz triangular and 1 kHz, 100 Hz square waveforms. The frequency range is of importance for FTJ-based biological time scale neuromorphic circuits,^26,27^ where devices are expected to be switched on the order of several 100 Hz frequency. Also, in our measurement setup, square pulses with amplitude in the range of 5 V can be applied reliably without distortion for 1 kHz and lower frequencies. Considering these factors, the wake-up study is performed at 1 kHz and 100 Hz, which cover 1 order of magnitude in the frequency range. Each waveform mentioned earlier has a voltage amplitude of ±4.5 V, as shown in the schematic in Figure 1b. The *I*–*V* and *P*–*V* measurements performed before and after wake-up cycles for the four waveforms are shown in Figure 1c–f. Note that 2000 cycles with a 1 kHz triangular waveform are the typical wake-up cycling for our TiN–HZO–TiN capacitors.^12^ All of the measurements were carried out with 1 ms width bipolar triangular pulses (voltage sweep: 0 → 4.5 V → 0 → −4.5 V → 0), as shown in Figure 1b. In the pristine state, there is a low remnant polarization (*P*_R_) of 4–5 μC/cm^2^. The *P*–*V* loops are not pinched, clearly showing ferroelectric behavior. The switching current peak in the *I*–*V* measurements is broader for the positive voltage polarity (switching current peak extends above 4 V) than for the negative one (switching current peak minimum is around −4 V). In *P*–*V* measurements, this translates to a lower slope of the curve when switching from −*P*_R_ to +*P*_R_ than the one when switching from +*P*_R_ to −*P*_R_. This indicates a broader distribution of coercive voltages, with some domains not switched during the voltage sweep in the positive polarity, as their coercive voltage could even be above 4.5 V. The coercive voltage distribution for the two polarities has a higher asymmetry in comparison to a typical M–HZO–M stack. This is due to the dielectric–ferroelectric interface on one side, which could have domain pinning due to the presence of charge traps or oxygen vacancies, particularly for the domains whose polarization points toward Al_2_O_3_. Such an effect has been observed in TiN–HZO–TiN stacks where the bottom TiN electrode exhibit oxide formation.^28^ Thus, it may result in some domains having high coercive voltages (even above 4.5 V in our devices). As shown in Figure 1c, the wake-up effect upon cycling with a 1 kHz triangular waveform is observed to be very weak with a minor increase in the switching current peak and a corresponding low remnant polarization magnitude. After 2000 cycles, the coercive voltage distribution of domains does not seem to change much from the pristine state. However, when wake-up cycling is performed with a 100 Hz triangular waveform, as shown in Figure 1d, a considerable increase in the switching current peak from pristine to 1000 cycles is observed. The remnant polarization increases in magnitude to 2*P*_R_ ∼ 20 μC/cm^2^. The coercive voltage for positive polarity decreases, and the distribution of the coercive voltages of domains is narrower than that in the pristine state, as shown by the reduced width of the switching current peak. A further improvement in wake-up behavior is observed when field cycling is performed with the square waveform with a frequency of 1 kHz, as shown in Figure 1e, which further increases for 100 Hz (Figure 1f). The increase in the remnant polarization for square waveforms is much higher than those for the two triangular waveforms, and a very high remnant polarization of 2*P*_R_ ∼ 35 μC/cm^2^ for 1 kHz and 2*P*_R_ ∼ 39.5 μC/cm^2^ for 100 Hz is obtained. In both square waveforms, the coercive voltage distribution (indicated by switching current peak width) on the positive polarity is highly reduced compared to the pristine state as well as the woken-up state after cycling with the two triangular waveforms. This observation points to the switching of a much larger percentage of domains, as the coercive voltage of the majority of domains is now well below the maximum applied voltage (the switching current peak is achieved at nearly 3 V). The increased number of domains that now switch correspond to the domains whose coercive voltage was high (above 4 V in the pristine state). Therefore, we hypothesize that these domains have been depinned during square waveform cycling and that their coercive voltage has been reduced during wake-up cycles.^19,23^

**Figure 2:**
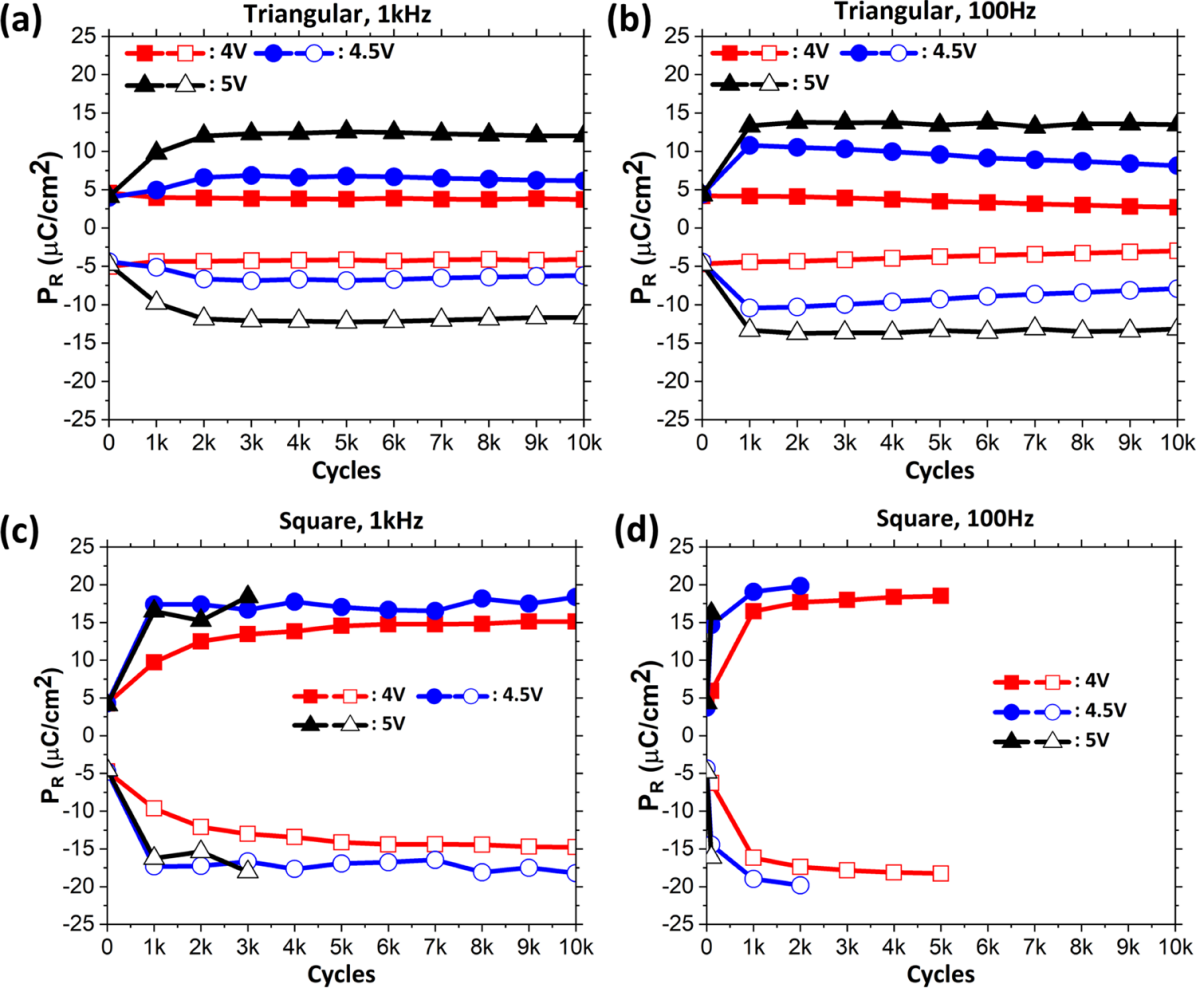
(a–d) Evolution of remnant polarization from pristine to 10^4^ cycles for the four wake-up waveforms for 4, 4.5, and 5 V cycling voltage amplitudes.

Figure 2a–d shows the evolution of remnant polarization (*P*_R_) from pristine to 10^4^ cycles for the four different wake-up waveforms presented in Figure 1 for 4, 4.5, and 5 V cycling. Wake-up is clearly not achieved for 4 V cycling for all waveforms and frequencies except the 100 Hz square waveform. Although the 4 V magnitude is slightly larger than the negative polarity coercive voltage distribution, it is clearly insufficient to overcome the coercive voltage distribution in positive polarity, as shown in Figure 1. Therefore, the wake-up is not achieved completely. A lower voltage amplitude of 3 V, which is clearly subcoercive, as shown in Figure S1 demonstrates an even lower increase in polarization with cycles. Although the polarization increases to 2*P*_R_ ∼ 32 μC/cm^2^ for the 4 V, 100 Hz square waveform, the number of cycles is limited to 5000 owing to oxide breakdown. Therefore, the 100 Hz square waveform leads to significant field stress on the oxide and is not favorable for achieving high remnant polarization post wake-up while maintaining higher endurance. Cycling with 4.5 V leads to better wake-up for all waveforms except for the 100 Hz square waveform, where the increased field leads to even lower endurance (2000 cycles). By further increasing the voltage to 5 V, it is observed that there is a significant improvement in the wake-up in 1 kHz triangular (2*P*_R_ ∼ 25 μC/cm^2^ at 3000 cycles) and 100 Hz triangular (2*P*_R_ ∼ 28 μC/cm^2^ at 3000 cycles) waveforms. Improvement in square waveforms is marginal but leads to a strong impact on endurance. Only 3000 cycles are achieved with 1 kHz square and 100 cycles with the 100 Hz square waveform. Therefore, 5 V leads to high field stress in square waveforms. As mentioned before, there is an asymmetry in coercive voltage distribution between the two polarities and a lower mean coercive voltage for negative polarity (around −3 V as indicated by the current peak in switching *IV* curves in Figure 1). Therefore, −5 V during each half-cycle of wake-up cycling leads to large field stress over the oxides and causes generation of defects, which ultimately leads to fatigue and device breakdown. Therefore, a balance between the field stress and wake-up for the two polarities is necessary and will be discussed in later sections.

**Figure 3:**
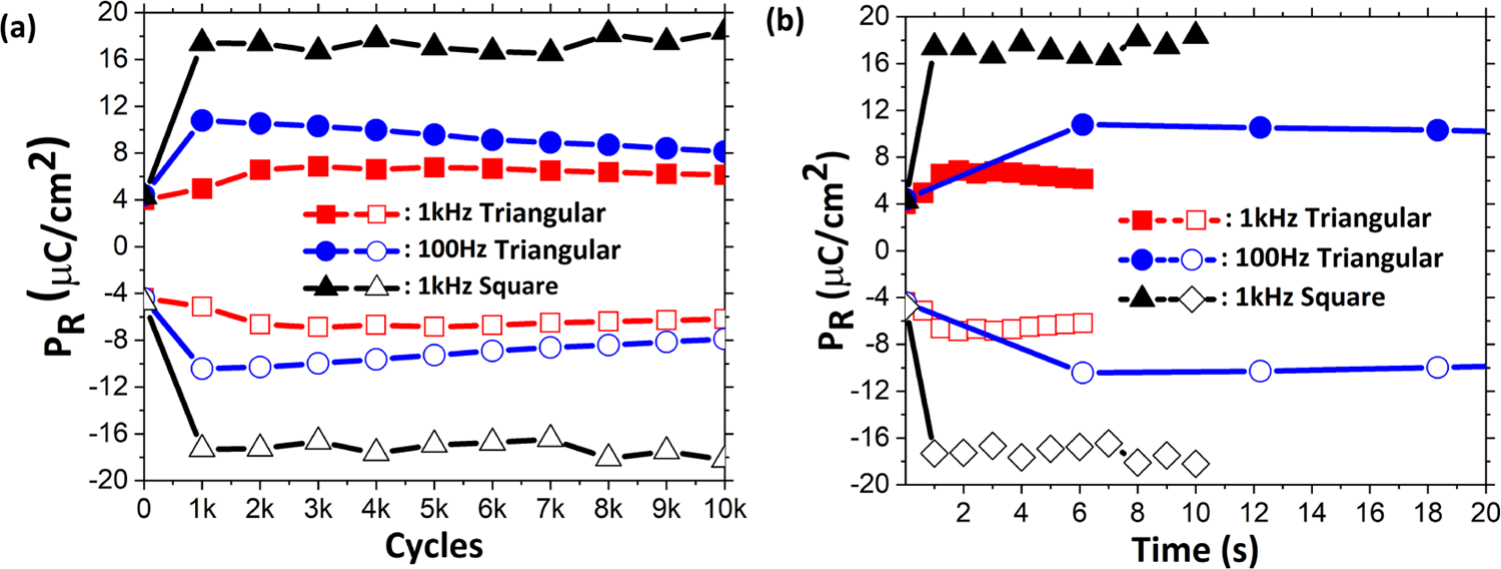
(a) Evolution of remnant polarization from pristine to 10^4^ cycles for the three wake-up waveforms. (b) Evolution of polarization with time. For square cycling, time is calculated as the number of cycles × pulse width, and for triangular cycling, the time for which the device experiences a voltage magnitude of above 1.75 V. This is the voltage at which the switching current in *I*–*V* starts to increase (Figure 1c,d), indicating switching of domains.

As observed in Figure 2, cycling with 4.5 V shows reasonable wake-up for most waveforms and allows up to 10^4^ cycles; it is considered for the comparison of wake-up between three waveforms: 1 kHz triangular, 100 Hz triangular, and 1 kHz square. Due to low endurance, 100 Hz square is not considered. The evolution of the remnant polarization *P*_R_ from pristine up to 10^4^ cycles for the three different wake-up sequences is compared as a summary in Figure 3a. Cycling with the 1 kHz square waveform leads to the highest value of 2*P*_R_ ∼ 35 μC/cm^2^ after only 1000 cycles, and remarkable stability of polarization is observed up to 10^4^ cycles. The 100 Hz and 1 kHz triangular cycles result in remnant polarization values of 2*P*_R_ ∼ 20 μC/cm^2^ (achieved after 1000 cycles) and 2*P*_R_ ∼ 14 μC/cm^2^ (achieved after 2000/3000 cycles), respectively. Furthermore, *P*_R_ starts to decrease after wake-up in both cases. Hence, it appears that the fatigue effect occurs at a lower number of cycles in the case of triangular cycling and is particularly strong for the 100 Hz triangular waveform. A similar effect is observed in subcoercive voltage cycling with 3 V for all three waveforms considered here (see Figure S1). This evident effect of fatigue due to insufficient wake-up is similar to the result by Li et al.^29^ In that work, insufficient wake-up, resulting from partial switching, due to lower voltage or shorter pulse width in the square waveform leads to an increase in the fatigue of the Pt/HZO/LSMO/STO stack. This was explained by an increased domain wall concentration and by pinning of the domains due to charged defects. The same mechanism might be at play here in triangular waveform cycling.

Starschich et al.^22^ reported that the duration of the applied electric field rather than the number of cycles is the primary factor responsible for wake-up in TiN–(Y doped) HfO_2_–TiN (M–FE–M) capacitor stacks. The work in ref (22), however, only involved the comparison of square waveforms of different frequencies (with symmetric negative and positive voltage amplitudes). The dependence of *P*_R_ with the duration of the applied electric field for the three waveforms is plotted in Figure 3b. For triangular waveforms, we consider the time during which the absolute value of the voltage was above 1.75 V. This is indeed the voltage at which the switching current in *I*–*V* curves starts to increase, as shown in Figure 1c–e. It is evident in Figure 3b that the remnant polarization is different for the three waveforms for any given duration after the wake-up. In the square waveform, the amplitude is constant in magnitude (±4.5 V) for the entire duration of the cycle. In triangular waveforms, however, the voltage is constantly changing, and the voltage is greater than 4 V for only a limited duration of each cycle. In the pristine state, the coercive voltage distribution extends up to −4 V for negative polarity (indicated by the minimum of the switching current peak in Figure 1c–e). Therefore, all of the domains can be assumed to switch from down to up during both the triangular and square waveforms, as the maximum amplitude applied is −4.5 V. However, for positive polarity, the coercive voltage distribution extends above 4.0 V. In the 1 kHz square waveform, the voltage is greater than 4.0 V for 0.5 ms in the positive polarity half-cycle. In the case of the 100 Hz triangular waveform, it is greater than 4.0 V for almost 0.55 ms (Figure S2). Even though both 100 Hz triangular and 1 kHz square waveforms have voltages larger than 4.0 V for a similar duration, the resultant *P*_R_ after wake-up is largely different for the two cases. The effective higher voltage in the square waveform (4.5 V for the entire 0.5 ms in the positive polarity half-cycle) seems to be an important factor in the wake-up process. The broader coercive voltage distribution for positive polarity in the pristine state (Figure 1c–f) is an indication of some domains being pinned pointing toward Al_2_O_3_ due to the presence of traps or vacancies near the Al_2_O_3_–HZO interface. Therefore, it is important to depin these domains during the positive polarity half-cycle to aid the wake-up. The root mean square (RMS) value of the voltage magnitude for the 100 Hz triangular waveform for voltages between 4.0 and 4.5 V (corresponding to the coercive voltages of some of the pinned domains) is 0.2885 V (=0.577 × 0.5 V). The 1 kHz square waveform RMS is 0.5 V. During switching, there is charge injection from the electrodes to charge traps (resulting in charging or discharging of the traps)^30^ and movement of oxygen vacancies under the applied field.^15,31^ Since the triangular waveform has a lower RMS voltage than the square one (hence a lower electric field), both the vacancy movement and charge injection are much reduced under the triangular waveform, even though both waveforms have the electric field applied for the same duration. These mechanisms are indicated in the schematic shown in Figure S3. Therefore, we assume that the effective higher voltage in square waveforms leads to better depinning of domains in positive polarity half-cycles (for domains with coercive voltage larger than 4.0 V) than triangular waveforms. This indicates that the charges at or around the Al_2_O_3_/HZO interface play a dominant role in the wake-up behavior of such bilayer FTJ stacks.

**Figure 4:**
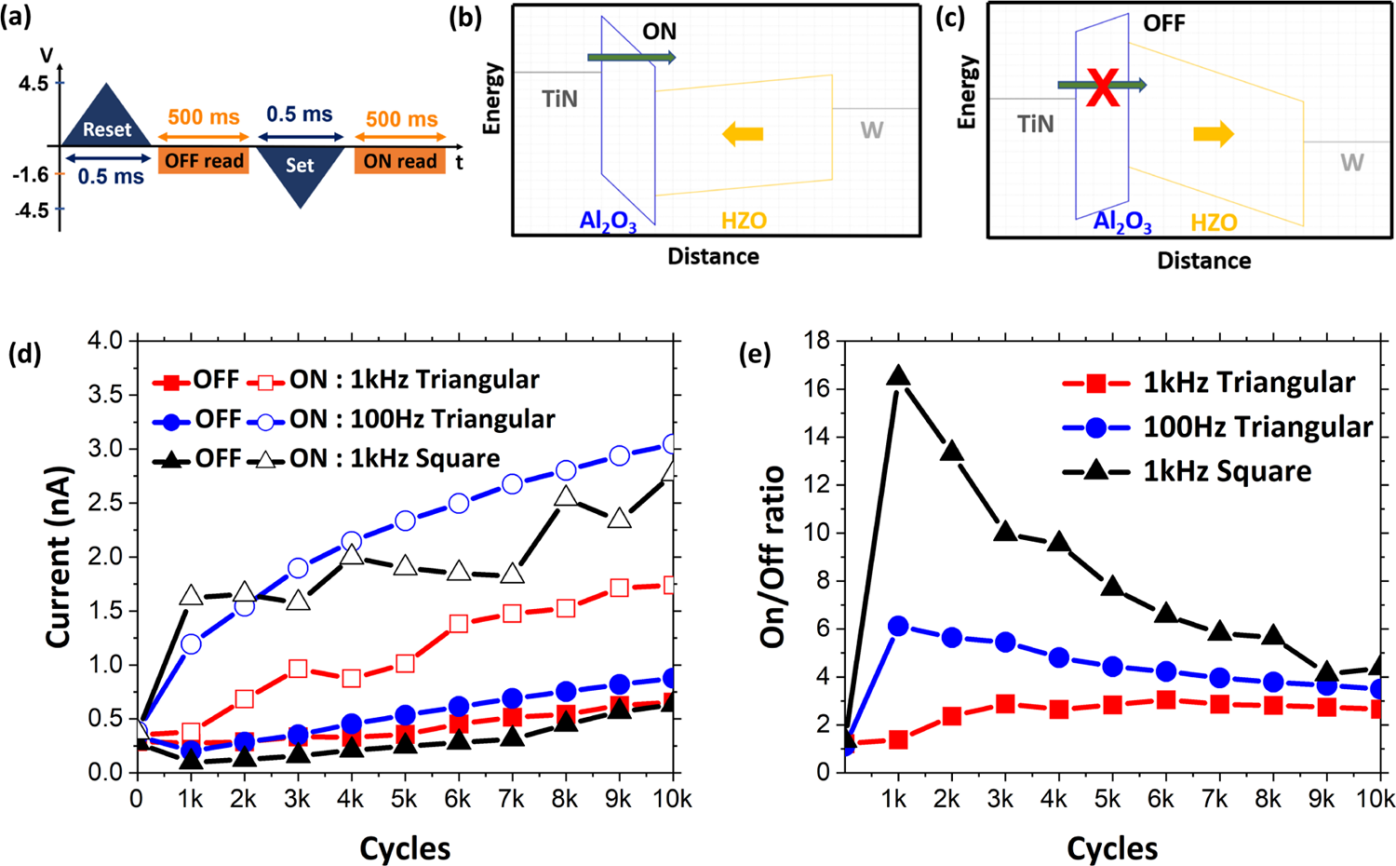
(a) Pulse sequence used for measuring OFF and ON state currents. Reset and set operations carried out with 0.5 ms width triangular pulses of +4.5 and −4.5 V, respectively. Read measurement is performed with a DC voltage of −1.6 V. Schematic of energy band diagram corresponding to (b) ON state (polarization toward Al_2_O_3_; −*P*_R_) and (c) OFF state (polarization toward the bottom W metal; +*P*_R_) under a read voltage bias. (d) Evolution of ON and OFF state currents with cycles for the three cycling waveforms. (e) Evolution of the ON/OFF ratio with cycles for the three different wake-up cycling waveforms.

To understand the wake-up cycling waveform impact on the memory functionality of FTJ devices, tunneling current (after reset and set operations) was measured up to 10^4^ cycles for each waveform sequence. Reset and set operations were performed with 0.5 ms width triangular pulses of +4.5 and −4.5 V magnitudes, respectively, after the application of the waveform for a given number of cycles. The corresponding OFF state current (measured after reset operation) and ON state current (measured after set operation) were recorded by application of a constant voltage of −1.6 V for 500 ms. The current reported is the average of the current measured in this duration. The measurement sequence is shown in Figure 4a. The schematics of the energy band diagram (based on the calculations of the similar stack in ref (14)) corresponding to the set and reset states under the applied read voltage are shown in Figure 4b,c respectively. The Al_2_O_3_ layer cannot fully compensate for the polarization-bound charges of ferroelectric HZO at the Al_2_O_3_/HZO interface. This results in a depolarization field in the HZO layer and results in an electric field in the Al_2_O_3_ layer even in the absence of an external field. Thus, the two polarization directions in the HZO layer have different band bending inside Al_2_O_3_. When the polarization points toward the Al_2_O_3_ layer, the electric field in the Al_2_O_3_ layer leads to a lowering of the conduction band of Al_2_O_3_. Therefore, electrons from the TiN top electrode can tunnel with a higher probability onto the conduction band of HZO, as shown in Figure 4b (ON state). The tunneling probability is largely reduced when the polarization points away from Al_2_O_3_ (OFF state), as shown in Figure 4c. The ON and OFF currents are governed by the depolarization field in the HZO layer, which is proportional to the value of remnant polarization^10^ (assuming the charge traps at the interface do not fully compensate for the bound charges^32^). Therefore, small *P*_R_ resulting from 1 kHz triangular waveform cycling leads to low ON current and to the lowest ON/OFF ratio, as shown in Figure 4d,e. In the case of 100 Hz triangular and 1 kHz square waveform cycling, the ON currents are larger. Owing to the largest *P*_R_ obtained for 1 kHz square waveform cycling, the largest ON currents and lowest OFF currents are obtained, resulting in the highest ON/OFF ratios of 17 among the three cycling waveforms. While all three waveforms show an increase of both ON and OFF currents with cycles, indicating generation of defects and onset of fatigue (Figure 4d), the OFF current corresponding to 1 kHz square cycling remains the lowest among the three waveforms. However, the reduction in the ON/OFF ratio in the square waveform after 1000 cycles is strong (from 17 at 1000 cycles to ∼5 at 10^4^ cycles). As this waveform results in high *P*_R_, it can be considered that the stack sustains the highest depolarization field among the three sequences. This depolarization field could be 0.5–1 MV/cm and generate defects over cycles,^15^ increasing the OFF state current and reducing the ON/OFF ratio. Despite this reduction, square waveform cycling results in a better ON/OFF ratio until 10^4^ cycles, a better *I*_ON_ – *I*_OFF_ value (which allows for more intermediate states), and better stability with cycles. It also highlights the delicate balance between high *P*_R_ and the fatigue effect in bilayer FTJ devices.

As discussed earlier, we observe that in the pristine state, the coercive voltage distribution is very broad (particularly for positive polarity). Some domains probably have a positive polarity coercive voltage larger than +4.5 V, due to domain pinning. From the measurements in Figure 2, it is evident that a higher effective voltage in the positive polarity half-cycle in 1 kHz square waveform cycling leads to switching of more domains that were pinned, and the highest *P*_R_ is obtained after only 1000 cycles. This is further improved for the 100 Hz square waveform (∼4.5 μC/cm^2^ higher *P*_R_). Increasing the voltage amplitude to ±5 V resulted in *P*_R_ ∼ 33 μC/cm^2^ after just 100 cycles (*P*_R_ for the 1 kHz square waveform is ∼35 μC/cm^2^ after 1000 cycles). However, from Figure 1c–f, we observe that the coercive voltage distribution in the negative polarity is narrower, and the mean coercive voltage is lower in magnitude; −5 V leads to very high field stress, so the devices break down after 100 cycles for 100 Hz and 3000 cycles for 1 kHz square ±5 V cycling operation. Therefore, different voltage magnitudes are necessary for the two polarities. Let us now discuss such measurements.

**Figure 5:**
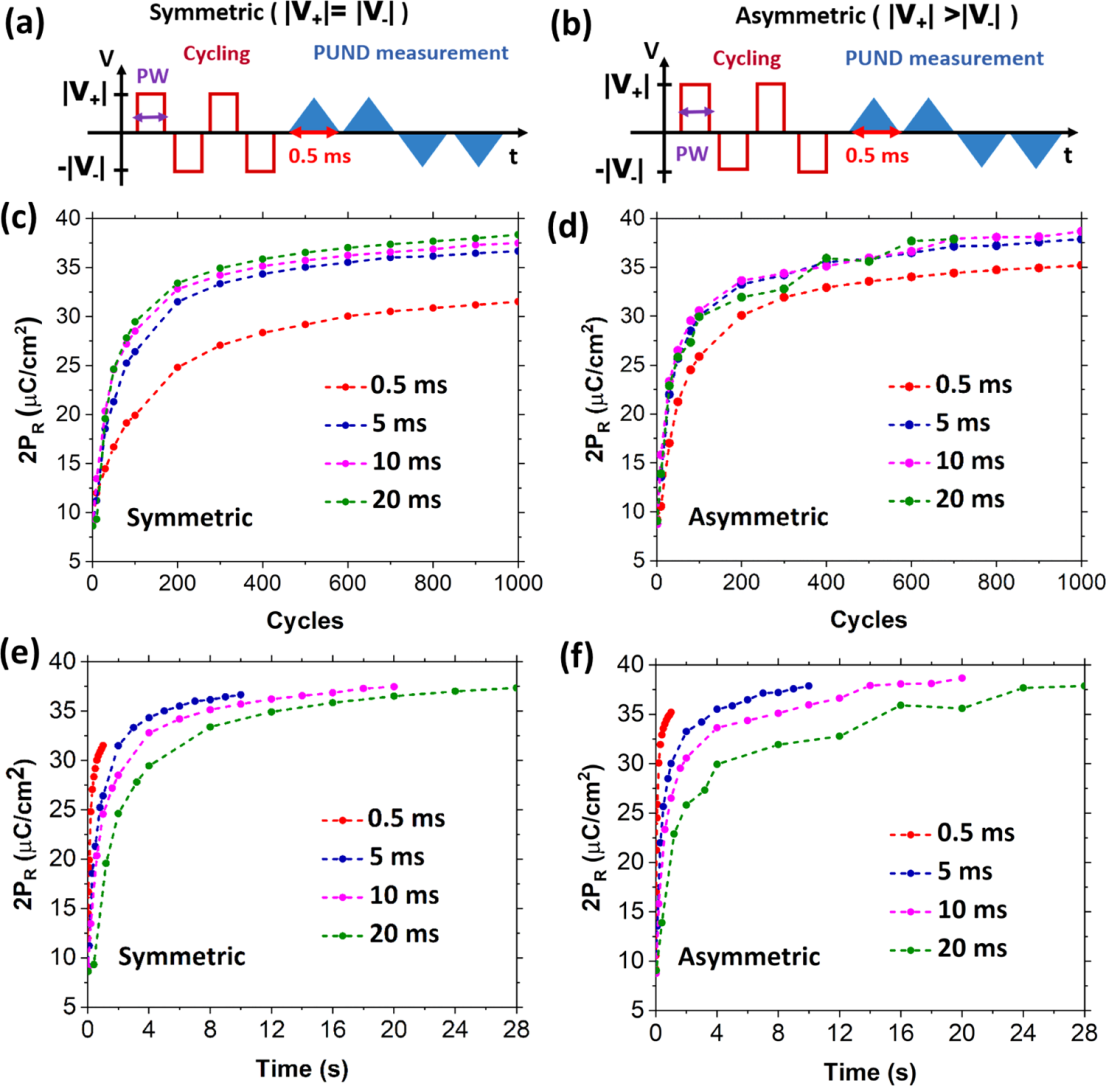
Schematic of the measurement sequence corresponding to cycling with the (a) symmetric pulse train, where the magnitudes of the square pulse for both polarities are equal, with |*V*_+_| = |*V*_–_| = 4.5 V, and (b) asymmetric pulse train, where the magnitudes for both polarities are unequal, with |*V*_+_| = 5 V and |*V*_–_| = 4.5 V. The time is calculated as: time = no. of cycles × 2 times pulse width. (c, d) Evolution of 2*P*_R_ from pristine to 1000 cycles for symmetric and asymmetric cycling pulses of varying pulse widths (0.5, 5, 10, and 20 ms). (e, f) Evolution of 2*P*_R_ with time for symmetric and asymmetric cycling pulses, respectively.

Our instrument does not have the functionality to apply a waveform with unequal magnitudes for the two polarities. Therefore, a cycling waveform was constructed from a series of alternating polarity square pulses (referred to as “pulse train”). The pulse train measurement sequence for equal voltage magnitudes for the two polarities is named “symmetric,” and different magnitudes are named “asymmetric,” as shown in the schematic in Figure 5a,b. Polarization of the devices with symmetric (*V*_+_ = 4.5 V, *V*_–_ = −4.5 V) and asymmetric (*V*_+_ = 5 V, *V*_–_ = −4.5 V) pulse train cycles is shown in Figure 5c,d, respectively, for different pulse widths of 0.5, 5, 10, and 20 ms. Figure 5c shows 2*P*_R_ improvement over cycles for the symmetric cycling pulse train for different pulse widths. With increasing pulse width, the wake-up (2*P*_R_ ∼ 32 μC/cm^2^) occurs with a lesser number of cycles. Particularly, there is a significant improvement between 0.5 and 5 ms. In the case of asymmetric pulse train cycling, the difference in wake-up between different pulse widths is minor, as shown in Figure 5d. By comparing 0.5 ms pulse width cycling for symmetric and asymmetric cases, we find that the wake-up occurs in a much lesser number of cycles (400) for the asymmetric case, while the symmetric one requires nearly 1000 cycles. There is a clear benefit of asymmetric cycling in terms of wake-up. The reduction in wake-up cycles could be the result of depinning, hence switching a larger number of domains during each positive polarity half-cycle.

To understand whether the wake-up is impacted by cycles or by the time of application of the electric field, Figure 5e,f shows the 2*P*_R_ value over the duration of the applied field (cycles × pulse width) for the symmetric and asymmetric pulse trains, respectively.^22^ For the symmetric pulse train, the remnant polarization reaches similar values after about 10 s for different pulse widths. However, for the asymmetric pulse train, the increase of the 2*P*_R_ value with time is very different for the different pulse widths. Particularly, even with the shortest pulse width (0.5 ms), a high 2*P*_R_ ∼ 35 μC/cm^2^ is reached. Considering the difference in evolution of *P*_R_ with time between symmetric and asymmetric pulses, it is clear that a higher positive voltage in asymmetric configuration “accelerates” the wake-up in the HZO layer.

**Figure 6:**
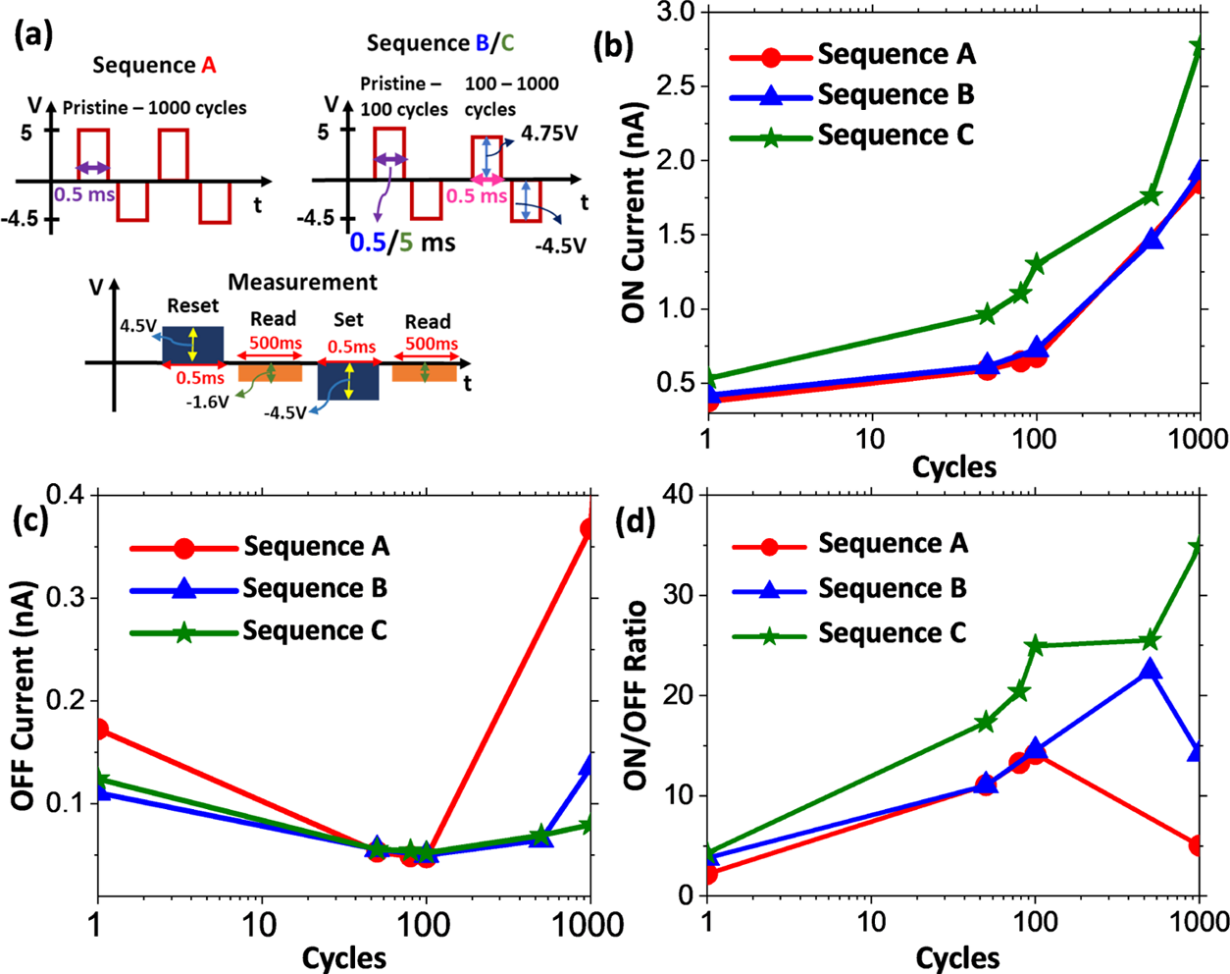
(a) Cycling and measurement sequences used for studying the impact of cycling pulses on the ON and OFF currents. Sequence A represents asymmetric cycling with +5 and −4.5 V with a pulse width of 0.5 ms from pristine to 1000 cycles. Sequence B corresponds to asymmetric cycling of +5 and −4.5 V with a pulse width of 0.5 ms from pristine to 100 cycles followed by asymmetric cycling of +4.75 and −4.5 V with a pulse width of 0.5 ms from 101 to 1000 cycles. Sequence C is similar to sequence B except for the pulse width of 5 ms during the first 100 cycles. Reset and set operations are performed with +4.5 and −4.5 V square pulses with a pulse width of 0.5 ms. The read operation of the OFF and ON states is performed with a DC bias of −1.6 V. (b, c) Evolution of the ON and OFF currents, respectively, from pristine to 1000 cycles for pulse sequences A, B, and C. (d) Evolution of the ON/OFF ratio from pristine to 1000 cycles for pulse sequences A, B, and C.

Since asymmetric pulse train cycling leads to higher remnant polarization in a lesser number of cycles, it is chosen for further study and optimization of ON and OFF currents and ON/OFF ratio. As observed in Figure 5d for the asymmetric case, the wake-up occurs around 200 cycles for a pulse width of 5 ms, and there is no further increase in remnant polarization with an increase in pulse width. Therefore, three pulse train sequences (named A, B, and C) consisting of different voltage magnitudes and two pulse widths (0.5 and 5 ms), as shown in Figure 6a, were further studied. The OFF and ON currents were measured after reset and set operations, respectively. Here, the reset and set operations were performed by square monopolar pulses of +4.5 and −4.5 V amplitudes, respectively. The read measurements for OFF and ON state currents were carried out by applying a constant voltage of −1.6 V. Sequence A shows a cycling pulse train waveform with +5 and −4.5 V amplitudes and a pulse width of 0.5 ms. In this sequence, the same pulse train is used to cycle the device from pristine to 1000 cycles. For sequence B, cycling from pristine to 100 cycles is performed with a square pulse train waveform with +5 and −4.5 V amplitudes and a pulse width of 0.5 ms. From 101 to 1000 cycles, the voltage amplitudes are changed to +4.75 and −4.5 V. Sequence C is similar to sequence B except that a pulse width of 5 ms (instead of 0.5 ms) is used for cycling from pristine to 100 cycles. Between sequences A and B, the only change is the reduction of the positive polarity cycling voltage amplitude to +4.75 V after 100 cycles. As observed in Figure 6b,c, this reduction has no impact on the ON current but has a strong beneficial effect on reducing the OFF current. The higher negative polarity voltage magnitude in sequence A possibly leads to defect generation and hence to the onset of fatigue in the FTJ already after 100 cycles. We hypothesize that the OFF current increase is a good indicator of fatigue and defect generation in the stacks. From band configuration in the OFF state (Figure 4c), an increase in OFF current should either be due to reduced polarization or an increase in defect states in the band gap of Al_2_O_3_ and/or HZO. As *P*_R_ continues to increase after 100 cycles in sequence A, the OFF state current increase could originate from defect generation. Hence sequences B and C have a delay in the onset of the fatigue effect. With a lower OFF current, the ON/OFF ratio is increased and remains higher for a larger number of cycles. Now in sequence C, the initial wake-up phase (from pristine to 100 cycles) is improved by increasing the pulse width from 0.5 to 5 ms. As shown in Figure 6d, such an increase leads to a significant improvement of the ON/OFF ratio and results in a high value of 35 at 1000 cycles. While single-layer HZO FTJs demonstrate a much higher ON/OFF ratio, we note that for similar bilayer stacks, the demonstrated ON/OFF ratios are in the range of 5–15. A comparison is provided in Table 1. Even after using a relatively low crystallization temperature of 400 °C, we are able to achieve an ON/OFF ratio of 35, thereby showing the advantage of optimized wake-up cycling for CMOS back-end compatible FTJ.

**Table 1:** Comparison of ON/OFF Ratios for Various HZO-Based Bilayer and Single-Layer FTJ Stacks

reference	FTJ structure	ON/OFF ratio
Bilayer		
this work	TiN/HZO/Al_2_O_3_/W	35
Max et al.^14^	TiN/HZO/Al_2_O_3_/TiN	10
Shekhawat et al.^33^	p-Ge/Al_2_O_3_/HZO/TiN	14
Ryu et al.^4^	p-Si/HZO/Al_2_O_3_/Ti/Au	5
Liu et al.^34^	Pt/ZrO_2_/HZO/Al_2_O_3_/HZO/ZrO_2_/Ti/Au	14.8
Bégon-Lours et al.^35^	TiN/TiO_2_/HZO/TiN	2
Bégon-Lours et al.^36^	TiN/WO*_X_*/HZO/TiN	7
Sulzbach et al.^37^	LSMO/HZO/Al_2_O_3_/Pt; LSMO/HZO/STO/Pt	700% (7); 390% (3.9)
		
Single Layer		
Ambriz-Vargas et al.^38^	TiN/Hf_0.5_Zr_0.5_O_2_/Pt	15
Prasad et al.^39^	LSMO/HfZrO_2_/Pt	135 (1 nm HZO), 10^5^ (2.5 nm HZO)
Sulzbach et al.^37^	LSMO/HZO/Pt	340% (3.4)
Sulzbach et al.^40^	LSMO/HZO/Pt	210% (2.1)
Ambriz-Vargas et al.^41^	Pt/HZO/Pt	20
Goh et al.^7^	W/HZO/TiN	16
Goh et al.^42^	p-Ge/HZO/TiN	20
Mikheev et al.^43^	p+Si/HZO/TiN	80

**Figure 7:**
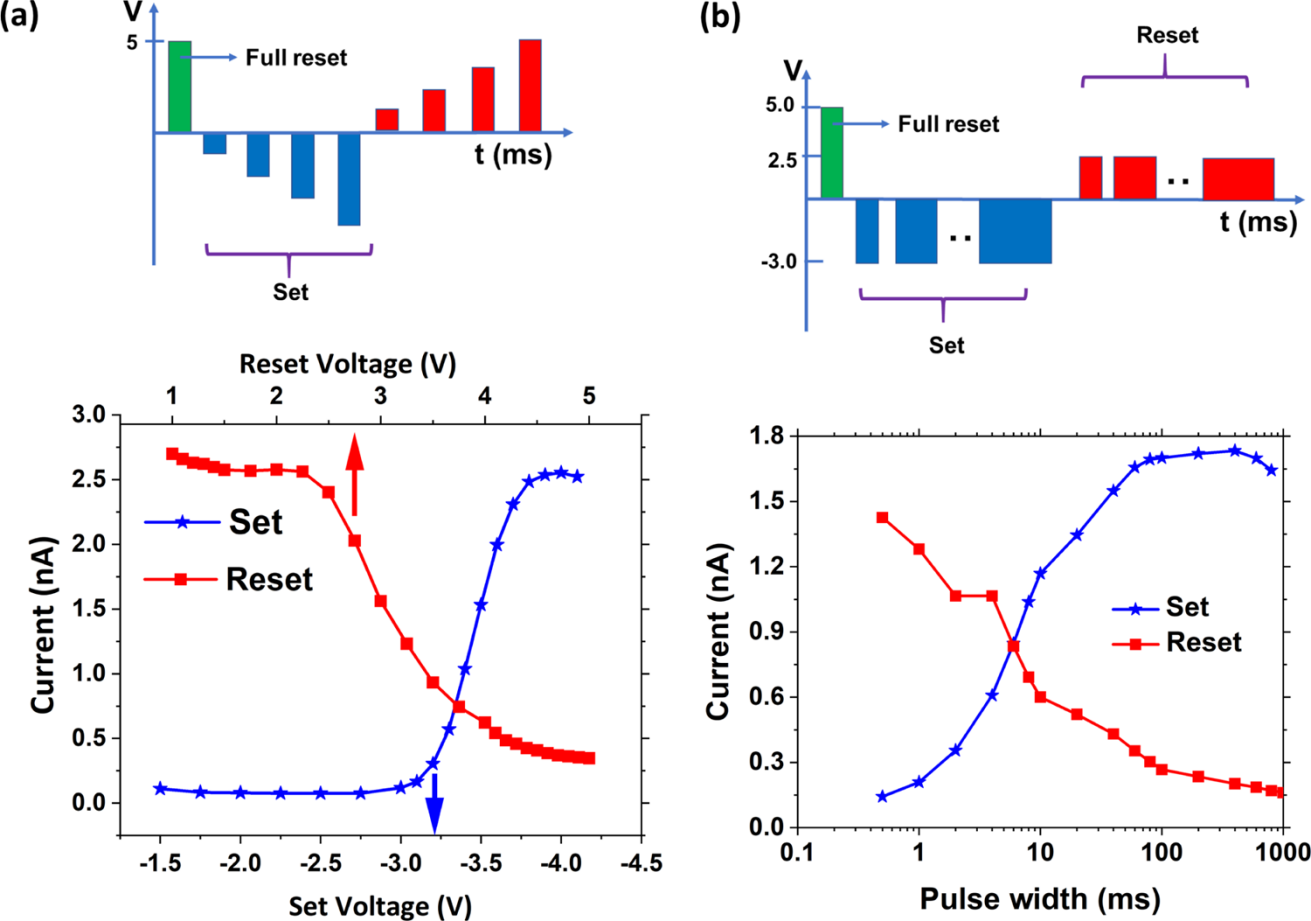
(a, b) Multiple resistance states in potentiation and depression attained through voltage amplitude and pulse width modification, respectively, after wake-up (1000 cycles), where the wake-up cycling operation was performed with optimized sequence C demonstrated in Figure 6. The schematics of pulse sequences used are shown above the graphs in (a) and (b). The full reset operation was carried out with a pulse width of 10 ms. In the sequence shown in (a), the set and reset pulses have a pulse width of 0.5 ms.

Finally, after obtaining the optimized sequence for a high ON/OFF ratio (sequence C with an ON/OFF current ratio of 35), the FTJs were woken up with this sequence, and partial switching of domains was carried out to demonstrate multiple resistance states, as shown in Figure 7. The partial switching was carried out with two sequences: (a) progressively increasing set or reset voltage amplitudes, with a fixed pulse width (Figure 7a) and (b) progressively increasing pulse width for the set or reset pulses with a fixed voltage amplitude (Figure 7b). The read current is measured after each partial switching reset and set pulses. Multiple well-separated current states are obtained. In Figure 7a, one can observe that at least six current states with a separation of more than 200 pA are observed for both reset and set operations. For reset operation, these states are observed between 2.5 and 4 V, and for set operation, between −3.25 and −3.75 V. Similarly, up to eight states are observed in set operation in Figure 7b between 1 and 100 ms pulse widths. A slight reduction in ON current is observed in Figure 7b for pulse widths >300 ms. In ref (25), it was shown that overcompensation of bound polarization charges by charging/discharging of traps leads to lower band bending and reduction of tunneling current (ON current in this case). Hence, for pulse widths >300 ms, the time scale is probably sufficient to charge/discharge a large number of traps, which contributes to the overcompensation of bound polarization charges. Through the partial switching measurements post wake-up, we demonstrate the advantage of the asymmetric pulse wake-up scheme for obtaining multiple well-separated current or resistance states needed for neuromorphic computing.

Our study highlights the impact of the inherent interface asymmetry of the ferroelectric layer in metal–dielectric–ferroelectric–metal FTJ devices on the device performance. In this FTJ architecture, the charge configuration at the ferroelectric/dielectric interface leads to different distributions of coercive voltages for the two polarization directions. A recent study^25^ has shown that an optimum level charge trap density is necessary at the ferroelectric/dielectric interface to stabilize both polarization directions. As we observed a large dependence of the remnant polarization on the wake-up cycling waveform, we can hypothesize that during wake-up cycling, charge traps necessary to stabilize the polarization are generated either by ion movement or field-induced trapping or detrapping. Therefore, tuning the voltage amplitude and pulse width of the cycling waveform is necessary to stabilize higher remnant polarization. The increase in remnant polarization translates directly to the increase of the ON/OFF ratio, which is consistent with observations on a similar stack in ref (24).

## Conclusions

Our study demonstrates that the cycling waveform has a large impact on the wake-up and resulting remnant polarization in TiN–Al_2_O_3_–HZO–W FTJ devices. Owing to the largely different interfaces (dielectric/ferroelectric and ferroelectric/metal) in such M–DE–FE–M stacks, strong domain pinning can occur for one of the polarization directions. We show that the wake-up effect depends strongly on the ability of the cycling waveform to depin the domains. Compared to triangular waveforms, square waveforms enable larger *P*_R_ after wake-up. It is further shown that different voltage magnitudes are necessary for switching the two polarization directions to reach the highest remnant polarization in the stack. Using an asymmetric waveform for field cycling and by changing the pulse width, we increase the ON/OFF ratio of the FTJ from ∼5 up to ∼35. Utilizing this optimized scheme, we demonstrate multiple well-separated resistance states in the FTJs. Our results emphasize the necessity of optimizing the cycling pulse scheme to attain higher ON/OFF ratios, which is essential for well separated multiple resistance states for neuromorphic computing.

## Supporting Information Available

Impact of cycling with triangular and square pulses of 1 kHz or 100 Hz frequency and 3 V magnitude; comparison of pulse shape between 100 Hz triangular and 1 kHz square cycling pulses; and schematic explaining the mechanisms leading to different wake-up in square and triangular waveforms (PDF)

## References

[ref1] Böscke T. S.; Müller J.; Bräuhaus D.; Schröder U.; Böttger U. Ferroelectricity in hafnium oxide thin films. Appl. Phys. Lett. 2011, 99, 102903 10.1063/1.3634052.

[ref2] Müller J.; Böscke T. S.; Schröder U.; Mueller S.; Bräuhaus D.; Böttger U.; Frey L.; Mikolajick T. Ferroelectricity in simple binary ZrO_2_ and HfO_2_. Nano Lett. 2012, 12, 4318–4323. 10.1021/nl302049k. 22812909

[ref3] Mikolajick T.; Schroeder U.; Slesazeck S. The Past, the Present, and the Future of Ferroelectric Memories. IEEE Trans. Electron Devices 2020, 67, 1434–1443. 10.1109/TED.2020.2976148.

[ref4] Ryu H.; Wu H.; Rao F.; Zhu W. Ferroelectric Tunneling Junctions Based on Aluminum Oxide/Zirconium-Doped Hafnium Oxide for Neuromorphic Computing. Sci. Rep. 2019, 9, 20383 10.1038/s41598-019-56816-x. 31892720 PMC6938512

[ref5] Mo F.; Tagawa Y.; Saraya T.; Hiramoto T.; Kobayashi M. In Scalability Study on Ferroelectric-HfO_2_ Tunnel Junction Memory Based on Non-equilibrium Green Function Method, 19th Non-Volatile Memory Technology Symposium (NVMTS), 2019; pp 1–5.

[ref6] Max B.; Hoffmann M.; Mulaosmanovic H.; Slesazeck S.; Mikolajick T. Hafnia-Based Double-Layer Ferroelectric Tunnel Junctions as Artificial Synapses for Neuromorphic Computing. ACS Appl. Electron. Mater. 2020, 2, 4023–4033. 10.1021/acsaelm.0c00832.

[ref7] Goh Y.; Hwang J.; Lee Y.; Kim M.; Jeon S. Ultra-thin Hf_0.5_Zr_0.5_O_2_ thin-film-based ferroelectric tunnel junction via stress induced crystallization. Appl. Phys. Lett. 2020, 117, 242901 10.1063/5.0029516.

[ref8] Cheema S. S.; Kwon D.; Shanker N.; Reis R. D.; Hsu S. L.; Xiao J.; Zhang H.; Wagner R.; Datar A.; McCarter M. R.; Serrao C. R.; Yadav A. K.; Karbasian G.; Hsu C. H.; Tan A. J.; Wang L. C.; Thakare V.; Zhang X.; Mehta A.; Karapetrova E.; Chopdekar R. V.; Shafer P.; Arenholz E.; Hu C.; Proksch R.; Ramesh R.; Ciston J.; Salahuddin S. Enhanced ferroelectricity in ultrathin films grown directly on silicon. Nature 2020, 580, 478–482. 10.1038/s41586-020-2208-x. 32322080

[ref9] Chernikova A.; Kozodaev M.; Markeev A.; Negrov D.; Spiridonov M.; Zarubin S.; Bak O.; Buragohain P.; Lu H.; Suvorova E.; Gruvermann A.; Zenkevich A. Ultrathin Hf_0.5_Zr_0.5_O_2_ Ferroelectric Films on Si. ACS Appl. Mater. Interfaces 2016, 8, 7232–7237. 10.1021/acsami.5b11653. 26931409

[ref10] Zhuravlev M. Y.; Wang Y.; Maekawa S.; Tsymbal E. Y. Tunneling electroresistance in ferroelectric tunnel junctions with a composite barrier. Appl. Phys. Lett. 2009, 95, 052902 10.1063/1.3195075.

[ref11] Max B.; Hoffmann M.; Slesazeck S.; Mikolajick T. Ferroelectric Tunnel Junctions Based on Ferroelectric-Dielectric Hf_0.5_Zr_0.5_O_2_/Al_2_O_3_ Capacitor Stacks, 48th European Solid-State Device Research Conference IEEE, 2018; pp 142–145.

[ref12] Deshpande V.; Nair K. S.; Holzer M.; Banerjee S.; Dubourdieu C. CMOS back-end-of-line compatible ferroelectric tunnel junction devices. Solid State Electronics 2021, 186, 108054 10.1016/j.sse.2021.108054.

[ref13] Jiang A. Q.; Lee H. J.; Kim G. H.; Hwang C. S. The Inlaid Al_2_O_3_ Tunnel Switch for Ultrathin Ferroelectric Films. Adv. Mater. 2009, 21, 2870–2875. 10.1002/adma.200802924.

[ref14] Max B.; Hoffmann M.; Slesazeck S.; Mikolajick T. Direct correlation of ferroelectric properties and memory characteristics in ferroelectric tunnel junctions. IEEE J. Electron Devices Soc. 2019, 7, 1175–1181. 10.1109/JEDS.2019.2932138.

[ref15] Pešić M.; Fengler F. P. G.; Larcher L.; Padovani A.; Schenk T.; Grimley E. D.; Sang X.; LeBeau J. M.; Slesazeck S.; Schroeder U.; Mikolajick T. Physical Mechanisms behind the Field-Cycling Behavior of HfO_2_-Based Ferroelectric Capacitors. Adv. Funct. Mater. 2016, 26, 4601–4612. 10.1002/adfm.201600590.

[ref16] Jiang P.; Luo Q.; Xu X.; Gong T.; Yuan P.; Wang Y.; Gao Z.; Wie W.; Tai L.; Lv H. Wake-Up Effect in HfO_2_-Based Ferroelectric Films. Adv. Electron. Mater. 2021, 7, 2000728 10.1002/aelm.202000728.

[ref17] Fields S. S.; Smith S. W.; Ryan P. J.; Jaszewski S. T.; Brummel I. A.; Salanova A.; Esteves G.; Wolfley S. L.; Henry M. D.; Davids P. S.; Ihlefeld J. F. Phase-Exchange-Driven Wake-Up and Fatigue in Ferroelectric Hafnium Zirconium Oxide Films. ACS Appl. Mater. Interfaces 2020, 12, 26577–26585. 10.1021/acsami.0c03570. 32410447

[ref18] Lederer M.; Olivio R.; Lehninger D.; Abdulazhanov S.; Kämpfe T.; Kirbach S.; Mart C.; Seidel K.; Eng L. M. On the Origin of Wake-Up and Antiferroelectric-Like Behavior in Ferroelectric Hafnium Oxide. Phys. Status Solidi RRL 2021, 15, 2100086 10.1002/pssr.202100086.

[ref19] Fengler F. P. G.; Hoffmann M.; Slesazeck S.; Mikolajick T.; Schroeder U. On the relationship between field cycling and imprint in ferroelectric Hf_0.5_Zr_0.5_O_2_. J. Appl. Phys. 2018, 123, 204101 10.1063/1.5026424.

[ref20] Kim H. J.; Park M. H.; Kim Y. J.; Lee Y. H.; Moon T.; Kim K. D.; Hyun S. D.; Hwang C. S. A study on the wake-up effect of ferroelectric Hf_0.5_Zr_0.5_O_2_ films by pulse-switching measurement. Nanoscale 2016, 8, 1383–1389. 10.1039/C5NR05339K. 26511062

[ref21] Walters G. H. Scaling and Design of Thin Film Ferroelectric Hafnium Oxide for Memory and Logic Devices. Doctoral Dissertation, University of Florida, 2020.

[ref22] Starschich S.; Menzel S.; Böttger U. Evidence for oxygen vacancies movement during wake-up in ferroelectric hafnium oxide. Appl. Phys. Lett. 2016, 108, 032903 10.1063/1.4940370.

[ref23] Chouprik A.; Spiridonov M.; Zarubin S.; Kirtaev R.; Mikheev V.; Lebedinskii Y.; Zakharchenko S.; Negrov D. Wake-Up in a Hf_0.5_Zr_0.5_O_2_ Film: A Cycle-by-Cycle Emergence of the Remnant Polarization via the Domain Depinning and the Vanishing of the Anomalous Polarization Switching. ACS Appl. Electron. Mater. 2019, 1, 275–287. 10.1021/acsaelm.8b00046.

[ref24] Lancaster S.; Mikolajick T.; Slesazeck S. A multi-pulse wakeup scheme for on-chip operation of devices based on ferroelectric doped HfO_2_ thin films. Appl. Phys. Lett. 2022, 120, 022901 10.1063/5.0078106.

[ref25] Fontanini R.; Segatto M.; Nair K. S.; Holzer M.; Driussi F.; Häusler I.; Koch C. T.; Dubourdieu C.; Deshpande V.; Esseni D. Charge-Trapping-Induced Compensation of the Ferroelectric Polarization in FTJs: Optimal Conditions for a Synaptic Device Operation. IEEE Trans. Electron Devices 2022, 69, 3694–3699. 10.1109/TED.2022.3175684.

[ref26] Covi E.; Donati E.; Liang X.; Kappel D.; Heidari H.; Payvand M.; Wang W. Adaptive Extreme Edge Computing for Wearable Devices. Front. Neurosci. 2021, 15, 611300 10.3389/fnins.2021.611300. 34045939 PMC8144334

[ref27] Gibertini P.; Fehlings L.; Lancaster S.; Duong Q. T.; Mikolajick T.; Dubourdieu C.; Slesazeck S.; Covi E.; Deshpande V. A Ferroelectric Tunnel Junction-based Integrate-and-Fire Neuron. 2022, arXiv:2211.02598. arXiv.org e-Print archive. https://arxiv.org/abs/2211.02598 (accepted at 29th International Conference on Circuits and Systems 2022).

[ref28] Luo Y. C.; Hur J.; Wang P.; Khan A. I.; Yu S. Non-volatile, small-signal capacitance in ferroelectric capacitors. Appl. Phys. Lett. 2020, 117, 073501 10.1063/5.0018937.

[ref29] Li X.; Li C.; Xu Z.; Li Y.; Yang Y.; Hu H.; Jiang Z.; Wang J.; Ren J.; Zheng C.; Lu C.; Wen Z. Ferroelectric Properties and Polarization Fatigue of La:HfO_2_ Thin-Film Capacitors. Phys. Status Solidi RRL 2021, 15, 2000481 10.1002/pssr.202000481.

[ref30] Lancaster S.; Lomenzo P. D.; Engl M.; Xu B.; Mikolajick T.; Schroeder U.; Slesazeck S. Investigating charge trapping in ferroelectric thin films through transient measurements. Front. Nanotechnol. 2022, 939822 10.3389/fnano.2022.939822.

[ref31] Chen J.; Jin C.; Yu X.; Liu Y.; Chen B.; Cheng R.; Han G.; et al. Impact of oxygen vacancy on ferroelectric characteristics and its implication for wake-up and fatigue of HfO_2_-based thin films. IEEE Trans. Electron Devices 2022, 69, 5297–5301. 10.1109/TED.2022.3190256.

[ref32] Si M.; Lyu X.; Ye P. D. On the Ferroelectric Polarization Switching of Hafnium Zirconium Oxide in Ferroelectric/Dielectric Stack. ACS Appl. Electron. Mater. 2019, 1, 745–751. 10.1021/acsaelm.9b00092.

[ref33] Shekhawat A.; Walters G.; Yang N.; Guo J.; Nishida T.; Moghaddam S. Data retention and low voltage operation of Al_2_O_3_/Hf_0.5_Zr_0.5_O_2_ based ferroelectric tunnel junctions. Nanotechnology 2020, 31, 39LT01 10.1088/1361-6528/ab9cf7. 32541100

[ref34] Liu Y.; Cao Y.; Zhu H.; Ji L.; Chen L.; Sun Q.; Zhang D. W. HfZrO_x_-Based Ferroelectric Tunnel Junction with Crested Symmetric Band Structure Engineering. IEEE Electron Device Lett. 2021, 42, 1311–1314. 10.1109/LED.2021.3102226.

[ref35] Bégon-Lours L.; Halter M.; Popoff Y.; Offrein B. J. Ferroelectric, Analog Resistive Switching in Back-End-of-Line Compatible TiN/HfZrO_4_/TiO_x_ Junctions. Phys. Status Solidi RRL 2021, 15, 2000524 10.1002/pssr.202000524.

[ref36] Bégon-Lours L.; Halter M.; Puglisi F. M.; Benatti L.; Falcone D. F.; Popoff Y.; Pineda D. D.; Sousa M.; Offrein B. J. Scaled, Ferroelectric Memristive Synapse for Back-End-of-Line Integration with Neuromorphic Hardware. Adv. Electron. Mater. 2022, 8, 2101395 10.1002/aelm.202101395.

[ref37] Sulzbach M. C.; Estandía S.; Gàzquez J.; Sánchez F.; Fina I.; Fontcuberta J. Blocking of Conducting Channels Widens Window for Ferroelectric Resistive Switching in Interface-Engineered Hf_0.5_Zr_0.5_O_2_ Tunnel Devices. Adv. Funct. Mater. 2020, 30, 2002638 10.1002/adfm.202002638.

[ref38] Ambriz-Vargas F.; Kolhatkar G.; Broyer M.; Youssef A. H.; Nouar R.; Sarkissian A.; Thomas R.; Yańez C. G.; Gauthier M. A.; Ruediger A. A Complementary Metal Oxide Semiconductor Process-Compatible Ferroelectric Tunnel Junction. ACS Appl. Mater. Interfaces 2017, 9, 13262–13268. 10.1021/acsami.6b16173. 28368099

[ref39] Prasad B.; Thakare V.; Kalitsov A.; Zhang Z.; Terris B.; Ramesh R. Large Tunnel Electroresistance with Ultrathin Hf_0.5_Zr_0.5_O_2_ Ferroelectric Tunnel Barriers. Adv. Electron. Mater. 2021, 7, 2001074 10.1002/aelm.202001074.

[ref40] Sulzbach M. C.; Tan H.; Estandía S.; Gàzquez J.; Sánchez F.; Fina I.; Fontcuberta J. Polarization and Resistive Switching in Epitaxial 2 nm Hf_0.5_Zr_0.5_O_2_ Tunnel Junctions. ACS Appl. Electron. Mater. 2021, 3, 3657–3666. 10.1021/acsaelm.1c00604.

[ref41] Ambriz-Vargas F.; Kolhatkar G.; Thomas R.; Nouar R.; Sarkissian A.; Yanez C. G.; Gauthier M. A.; Ruediger A. Tunneling electroresistance effect in a Pt/Hf_0.5_Zr_0.5_O_2_/Pt structure. Appl. Phys. Lett. 2017, 110, 093106 10.1063/1.4977028.

[ref42] Goh Y.; Jeon S. Enhanced tunneling electroresistance effects in HfZrO-based ferroelectric tunnel junctions by high-pressure nitrogen annealing. Appl. Phys. Lett. 2018, 113, 052905 10.1063/1.5040031.

[ref43] Mikheev V.; Chouprik A.; Lebedinskii Y.; Zarubin S.; Markeev A. M.; Zenkevich A. V.; Negrov D. Memristor with a ferroelectric HfO_2_ layer: In which case it is a ferroelectric tunnel junction. Nanotechnology 2020, 31, 215205 10.1088/1361-6528/ab746d. 32040945

