# Supplementary material for: Cycling Waveform Dependent Wake-Up and ON/OFF Ratio in Al2O3/Hf0.5Zr0.5O2 Ferroelectric Tunnel Junction Devices
Source: ACS Appl Electron Mater. 2023 Mar 10;5(3):1478–88. doi: 10.1021/acsaelm.2c01492 (PMC10064796; doi:10.1021/acsaelm.2c01492)
Supplement: Supplementary file 1 — el2c01492_si_001.pdf [file el2c01492_si_001.pdf]

Supporting information

Cycling waveform dependent wake-up and ON/OFF ratio in $Al_2O_3/Hf_{0.5}Zr_{0.5}O_2$ ferroelectric tunnel junction devices

Keerthana Shajil Nair[1,2], Marco Holzer[1,2], Catherine Dubourdieu[1,2, a,*], and Veeresh Deshpande[1, b,*]

[1]Helmholtz-Zentrum-Berlin für Materialien und Energie, Institute Functional Oxides for Energy Efficient Information Technology, Hahn-Meitner Platz 1, 14109 Berlin, Germany

[2]Freie Universität Berlin, Physical Chemistry, Arnimallee 22, 14195 Berlin, Germany

* Corresponding authors: a) catherine.dubourdieu@helmholtz-berlin.de; b) veeresh.deshpande@helmholtz-berlin.de

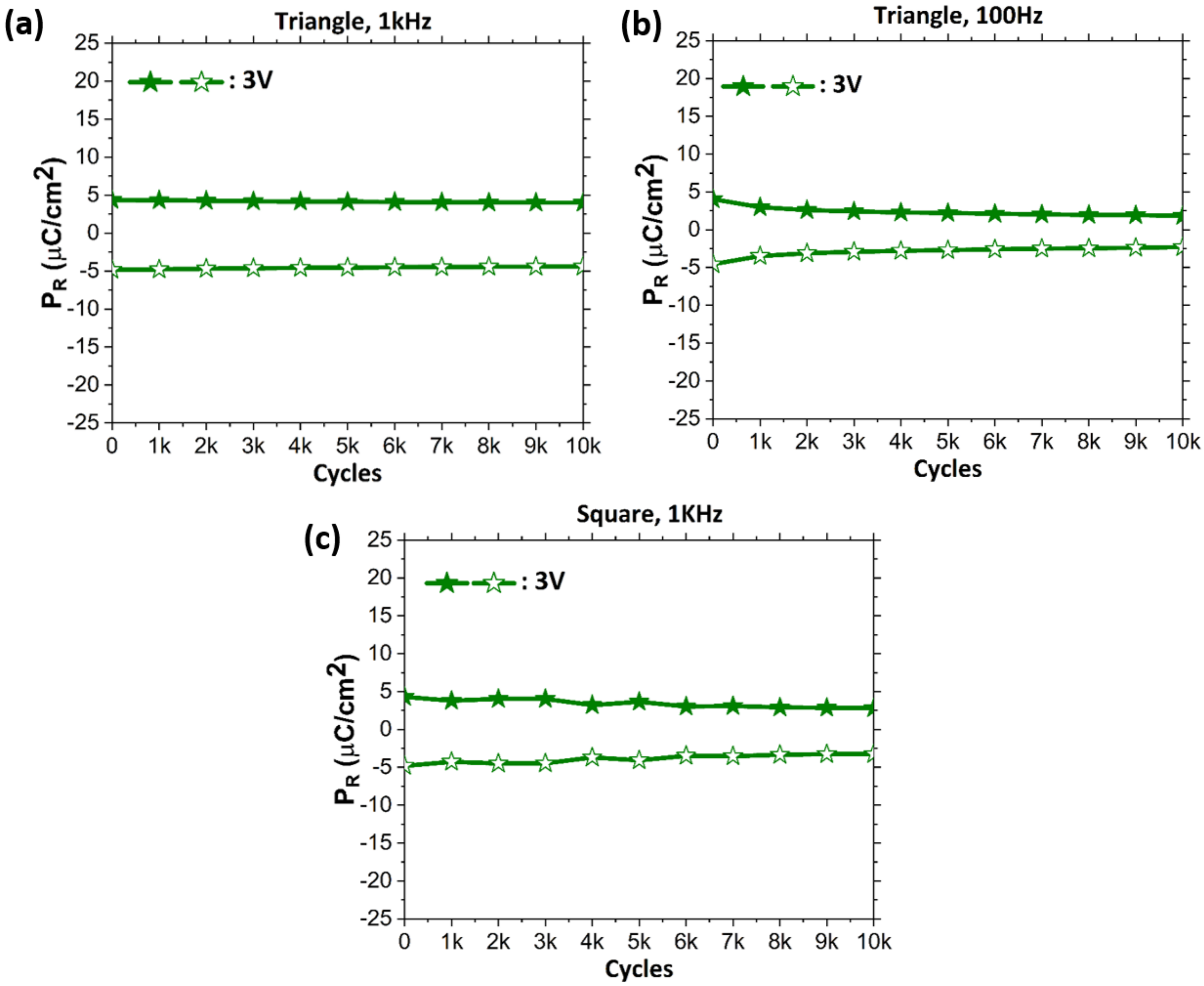

Figure S1. (a), (b) and (c) shows the evolution of remnant polarization from pristine to 10^4 cycles with cycling pulses of triangle 1kHz, triangle 100Hz and square 1kHz profiles respectively and 3V pulse amplitude.

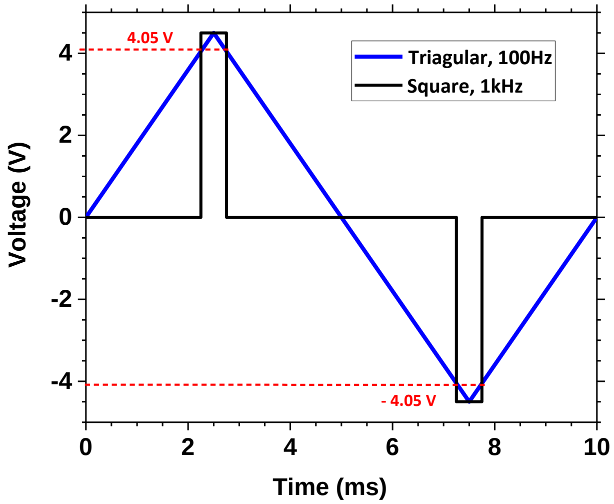

Figure S2. Comparison of the magnitude of voltage with time for positive and negative polarity half cycles for square 1 kHz and triangular 100 Hz waveforms. The half cycles of square waveform are shown only around the amplitude maxima of triangular 100 Hz wave for clear visibility. In square 1 kHz waveform, the amplitude is +4.5 V and -4.5 V for positive and negative polarity half cycles respectively. Each half cycle has a duration of 0.5 ms. For this duration, in triangular 100 Hz waveform, the amplitude is greater than 4.05 V for positive polarity half cycle and less than -4.05 V for negative polarity half cycle. Therefore, both the square 1 kHz and triangular 100 Hz waveforms have the magnitude of voltage amplitude greater than 4 V for 0.5 ms during each polarity half cycle.

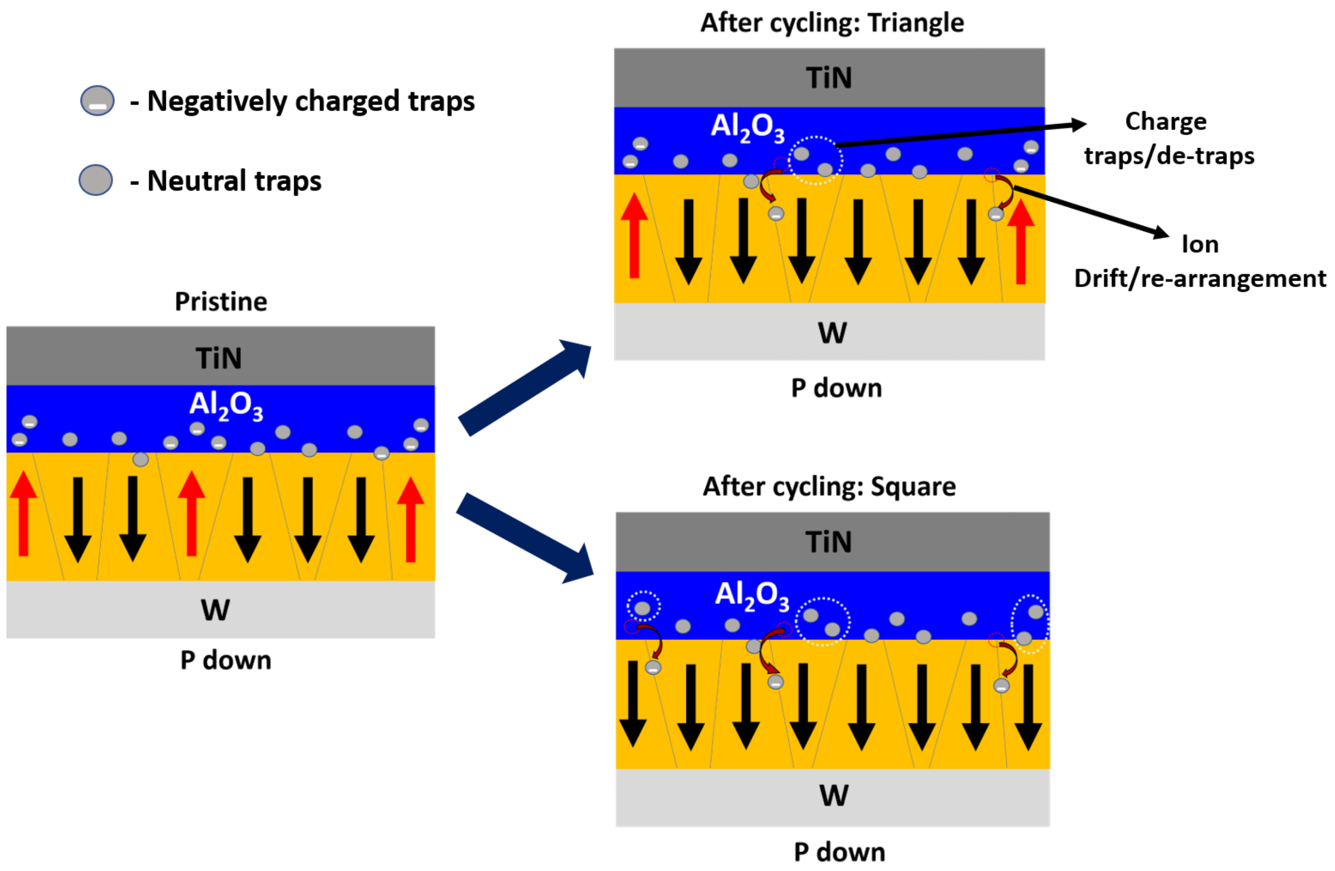

Figure S3. This schematic represents the mechanism behind higher remnant polarization after square cycling. In the pristine state, when we apply switching voltage to switch the polarization from P-up state to P-down state, not all the domains are able to switch to the P-down state. Some of the domains are pinned to P-up state due to the presence of negatively charged traps. In order to make these pinned domains take part in the P-down switching, these charges have to be neutralized by charge trapping/de-trapping or drift/re-arrange themselves in such a way that it enables the pinned domains to be switched. Both of these process are stronger (more charge trap/de-trapping or ion movement to longer distances) at higher electric fields. The effective higher voltage under positive polarity for 1 kHz square waveform compared to 100 Hz triangular therefore enables better wake-up with square waveform.
